# Systematic review of mass media interventions designed to improve public recognition of stroke symptoms, emergency response and early treatment

**DOI:** 10.1186/1471-2458-10-784

**Published:** 2010-12-23

**Authors:** Jan Lecouturier, Helen Rodgers, Madeleine J Murtagh, Martin White, Gary A Ford, Richard G Thomson

**Affiliations:** 1 Institute of Health and Society, Newcastle University, Newcastle upon Tyne, UK; 2 Institute for Ageing and Health (Stroke Research Group), Newcastle University, Newcastle upon Tyne, UK; 3 Medical and Social Care Education, Leicester University, Leicester, UK

## Abstract

**Background:**

Mass media interventions have been implemented to improve emergency response to stroke given the emergence of effective acute treatments, but their impact is unclear.

**Methods:**

Systematic review of mass media interventions aimed at improving emergency response to stroke, with narrative synthesis and review of intervention development.

**Results:**

Ten studies were included (six targeted the public, four both public and professionals) published between 1992 and 2010. Only three were controlled before and after studies, and only one had reported how the intervention was developed. Campaigns aimed only at the public reported significant increase in awareness of symptoms/signs, but little impact on awareness of need for emergency response. Of the two controlled before and after studies, one reported no impact on those over 65 years, the age group at increased risk of stroke and most likely to witness a stroke, and the other found a significant increase in awareness of two or more warning signs of stroke in the same group post-intervention. One campaign targeted at public and professionals did not reduce time to presentation at hospital to within two hours, but increased and sustained thrombolysis rates. This suggests the campaign had a primary impact on professionals and improved the way that services for stroke were organised.

**Conclusions:**

Campaigns aimed at the public may raise awareness of symptoms/signs of stroke, but have limited impact on behaviour. Campaigns aimed at both public and professionals may have more impact on professionals than the public. New campaigns should follow the principles of good design and be robustly evaluated.

## Background

Given the evidence of effectiveness of thrombolysis for acute stroke and of stroke units, stroke should be treated as a medical emergency in the same way as myocardial infarction [1-3]. However, many patients are seen too late to benefit from early treatment, often because of a lack of knowledge or awareness of stroke symptoms, or lack of emergency response to them, on the part of both the public and professionals [4]. Other factors such as a belief that the symptoms will subside or that nothing can be done may also play a part in delay to presentation at hospital [5]. Reviews of studies assessing levels of stroke knowledge and awareness among the general public, stroke patients and those at increased risk of stroke [6,7] have concluded that knowledge of stroke symptoms is generally poor and although most recognise the need for an emergency response this may not translate into action. A recent study reported that an adequate knowledge of stroke symptoms (i.e. three correct signs) is not associated with the intention to call emergency services in response to stroke [8].

A number of different interventions to improve knowledge of stroke symptoms and the appropriate action have been tested, for example, community stroke screening events, patient education programmes and mass media campaigns. A drawback of screening events and patient education programmes is they often target small numbers of people from a specific group such as those who have suffered, or are at risk of, a stroke when it is argued there is a need to target wider demographic groups [9] and those who may be a witness to a stroke [10]. Mass media interventions, although more costly, have the potential to reach a much larger audience.

Mass media interventions have been successful in reducing the use of tobacco [11], in improving road safety by reducing drink and driving [12], and increasing the use of safety belts [13]. However, in other health related areas they have had a small to moderate impact on behaviour change [14]. Considering acute myocardial infarction - similar to stroke in that the event should be regarded as an emergency - the success of mass media campaigns in changing behaviour at the onset of symptoms was mixed. Two reviews concluded that mass media interventions had little impact on reducing delay to presentation at hospital and the findings are difficult to interpret as most studies were methodologically flawed [15,16]. Neither review commented on the quality of, or theoretical base for, intervention development.

The evidence base for mass media campaigns more widely is itself limited; a Cochrane systematic review included 15 studies evaluating mass media campaigns designed to increase health service utilisation, all interrupted time series, and of variable methodological quality [17]. The authors concluded that, despite limitations, there was evidence that such campaigns may have "an important role in influencing the use of health care interventions" but that "further research... is needed on whether mass media coverage brings about appropriate use of services in those patients who will benefit most". It has been argued that adhering to the principles of effective campaign design has led to an increase in the success of mass media campaigns over the years [18]. The major principles of good design are as follows: gain an understanding of the target audience in terms of the problem behaviour, their preferred message and the most effective means of delivering that message, through exploratory research; use theory to identify the focus of the campaign message; to achieve maximum effectiveness, segment the audience to create groups with similar message preferences; design the message from the findings of the exploratory research and choose the channels most widely viewed by the target group; evaluate and monitor the process of campaign activities; use a rigorous design to evaluate the intervention such as time series and controlled before and after designs [18].

In England, as part of the national stroke strategy [19], the Department of Health recently implemented a national mass media campaign to promote public awareness of stroke symptoms and of the need for emergency response using the FAST (Face, Arm Speech, Time) test [20]. Implementation of this campaign begs an important question about the evidence base for the effectiveness of mass media campaigns in this specific area in changing knowledge of stroke (signs and symptoms) and, more importantly, behaviour (calling an emergency ambulance to ensure rapid access to treatment).

In light of the recent national stroke awareness campaign in the UK and the continued use of mass media campaigns in other countries it is timely to review the effectiveness and development of these interventions. Other reviews in the area of stroke education have focused on stroke prevention [21] or have included a combination of different types of interventions [22]. We were unable to identify any reviews examining the effectiveness and design of mass media interventions to improve knowledge of stroke symptoms and awareness of the need for an emergency response. In terms of reviewing the development of the intervention, we believe mass media campaigns are complex interventions - where often the aim is to change behaviour and improve knowledge - and should adhere to the structured development and evaluation as suggested by the MRC Framework [23].

The aims of this study are to:

• conduct a systematic review to assess the effectiveness of mass media campaigns in changing knowledge (stroke symptoms/signs and need for emergency access), behaviour (access to emergency services) or early treatment with thrombolysis.

• examine the methods and theoretical basis for development of the interventions using the MRC Framework guidance.

## Methods

### Search strategy

The Cochrane Stroke Group search terms for stroke [24] were used along with other terms developed, tested and then agreed by the study team and adapted for each database (Additional File 1). Searches were conducted in ten electronic databases (MEDLINE, EMBASE, CINAHL, Web of Knowledge, CSA Ilumina - ASSIA, Sociological Abstracts -, PsycInfo, ZETOC, AgeInfo and FRANCIS) from 1980 to 2010, the Cochrane Library (1980-2010), EPPI-Centre database and National Research Register. Manual searches through the reference lists of papers were also carried out.

### Inclusion and exclusion criteria

Primary studies in English evaluating the effectiveness of mass media interventions were included; where relevant, related papers were obtained to gather information on the methods of intervention development. Studies were selected according to the following criteria: (a) targeted groups: the general public aged 18 or over; (b) outcomes: knowledge of stroke symptoms and, awareness of the need for an emergency response, rates of acute stroke treatments, and time to presentation at hospital; (c) design: randomised controlled trials (RCTs), quasi-experimental studies, controlled (CBA) and uncontrolled before and after (BA) studies and interrupted time series (ITS). Interventions aimed solely at health professionals were excluded, but those that targeted both the general public and health professionals were included.

### Data extraction

Titles and abstracts were screened to identify studies of likely relevance and full papers obtained. A structured form was used to determine study inclusion. Two reviewers (JL, HR) extracted data from the final papers into structured tables. The results are presented as a narrative synthesis as the interventions varied in their format and presentation, were evaluated using different methods and outcomes, and included a range of study populations.

### Review of intervention development using the MRC Framework

Intervention development for each study was classified using the five key phases suggested in the MRC Framework for evaluating complex interventions [25] as follows.

Preclinical phase to identify the evidence to support the type of intervention: this could be from a systematic review or identifying or developing relevant theory

Phase 1 Modelling the processes and outcomes of the intervention

Phase 2 Exploratory trial to test out the intervention and outcome measures

Phase 3 Definitive RCT

Phase 4 Long term implementation

These phases were entered into a matrix. Each study was examined, using the information from all relevant published articles, to determine how the intervention was developed and evaluated; a summary of the process was recorded into the matrix under the appropriate phase. This enabled the team to examine across studies the extent to which the intervention development and evaluation was in line with the guidance recommended in the MRC Framework.

## Results

**Figure 1:**
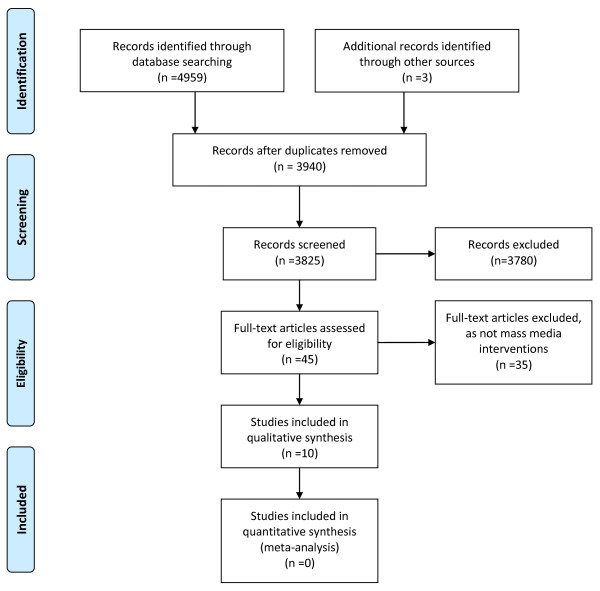
**PRISMA flow diagram of search results**.

Ten mass media intervention studies met the inclusion criteria and were included in the review (Figure 1). Six targeted the public [26-31] and four both public and professionals [32-35]. Study characteristics for the ten included studies are summarised in Additional File 2 and the results of evaluations in Additional File 3.

### Public only interventions

Six mass media campaigns with patient reported outcomes were reviewed [26-31]. Two studies used a controlled before and after design [30,31]. Campaign population coverage ranged from 80,000 to 5 million. The minimum duration of the intervention was two 10 week periods, the maximum 18 months. The longest period between the end of the campaign and outcome measurement was six months [27], the shortest one month [26]. The majority collected post-intervention data at only one point but one did so during and immediately following two campaigns and then six months later after the end of the second campaign [27]. One study [29] appears to have repeated their intervention in a different county in USA with the addition of the distribution of written educational materials and a control group [31].

#### Controlled before and after studies

In Ontario, Canada, Silver et al. [30] evaluated an 18 month campaign of continuous high level TV advertising, intermittent TV advertising and newspaper advertisements in each of three communities. A fourth community acted as a control and received only Heart and Stroke Foundation Public Service Announcements. Telephone interviews were conducted using random digit dialling; only residents aged 45 and over were interviewed. Quota sampling was used to ensure equal numbers of males and females and that at least one third of participants were aged 65 and over. The authors hoped to target men, older people and those in lower socioeconomic groups and hypothesised that television would be the most appropriate medium. The outcome of interest was knowledge of symptoms and signs, but not emergency response. At three months post-intervention there were significant improvements in groups exposed to both high intensity and low level television advertising but not to newspaper inserts. There was a significant decrease in the control group, an unexpected finding and one which the authors were unable to explain. The television interventions had a significant positive impact on younger (aged 45-64 years) respondents (p = 0.0001) and those with less than a secondary school education (p < 0.05), but did not increase knowledge of stroke symptoms in those aged 65 or over, the age group at greatest risk of stroke and most likely to witness a stroke. The low intensity television intervention was the most cost-effective method of raising awareness (measured by the gross rating points per percentage point change in outcome). This study was interesting in that, unlike the other studies, the team were able to examine the effectiveness of different media. One shortcoming was that the advertisements did not promote the need for an emergency response and numbers calling emergency services or attending hospital with suspected stroke were not measured. The study was underpowered and this precluded examination of the effects of past medical history or presence of stroke risk factors on stroke knowledge.

Fogle et al. [31] compared awareness of stroke warning signs and of the need for an emergency response between a group in Flathead County, exposed to 2 × 10 week mass media campaigns, and a control group in a Gallatin County, Montana. There was a two-month gap between the end of the first and beginning of the second campaign. The target audience for the campaign was those aged 45 years and over. The campaign consisted of four television and three radio advertisements and weekly newsprint advertisements for the duration of the intervention. The television advertisements covered: stroke warnings signs and the need for an emergency response; a three step stroke test on identifying a stroke; hospital staff reinforcing the need for an emergency response to stroke; and brain cell death following a stroke when treatment is delayed. The radio and newsprint advertisement ran along similar lines. Educational materials were posted to community doctors and pharmacies, churches and care homes for older people; a stroke information brochure and magnet were posted to households with residents in the target age group. The control county received no campaign information. Telephone surveys were conducted with residents aged 45 years and older in both counties, using sequential random digit dialling, before and after the campaign. It was not clear at what point pre- and post- campaign the telephone surveys were conducted. Following the campaign there was a statistically significant increase in those in the intervention group (regardless of sex or age) who could correctly identify two or more stroke warning signs (p ≤ 0.05): this increase was significant for those who had two or more self reported risk factors for stroke (p ≤ 0.05). There was no significant change in intention to call emergency services should they experience or witness a stroke. A higher proportion post-campaign would call emergency services if they experienced or witnessed the symptom of numbness (p ≤ 0.05) or any of the three symptoms (speech difficulties, numbness or paralysis) (p ≤ 0.05). There were no statistically significant increases of awareness of stroke warning signs or need for an emergency response in the control county group between the first and second surveys. There was a statistically significant increase in recall across all stroke campaign media (television, radio and newsprint) regarding stroke warning signs and the stroke test after the campaign but a smaller percentage recalled the advertisement for stroke test (television 32%; radio 16%; newsprint 21%) than the one for warning signs (television 71%; radio 32%; newsprint 43%). Although this campaign promoted an emergency response to stroke, as with the previous controlled study they did not measure whether this had any impact on behaviour when a stroke was suspected: in time to presentation to hospital or increased use of emergency services. Because of the design of the study the team are unable to demonstrate whether or not one particular media is more successful at increasing awareness of stroke symptoms.

#### Uncontrolled before and after studies

Becker et al. [26] assessed the impact of a five month campaign on Washington, USA, residents' level of stroke knowledge and on proposed action on witnessing a stroke. The five month campaign targeted people aged 65 years and over and consisted of: public service announcements and public interest stories on television; advertisements and stroke interest stories in newspapers; and public stroke screenings and flyers. Telephone interviews were conducted with residents who could speak English. Participants were selected using random digit dialling. One month after the end of a campaign, using both television and newspapers, respondents were more likely to know at least one symptom of stroke than one month before the campaign (p = 0.032). However, fewer (non-significant) would call emergency services if they witnessed a stroke, despite this being a major focus of the campaign. Post-intervention outcomes were collected at one month after the end of the campaign so little can be said about the impact of the intervention in the longer term. In addition, although the message was to promote an emergency response to stroke, only a change in a person's *intention *to act rather than an actual change in behaviour could be measured.

In Mainz, Germany Marx et al. [28] reported the impact of a mass media campaign of three months' duration. The intervention consisted of: billboard and poster advertisements with short slogans; stroke interest stories, slogans and interviews in local newspapers; stories, reports and interviews on television and radio; public events; flyers sent to every household; and a stroke guide distributed at the public events and through hospital and family doctors. Telephone surveys were conducted two months pre- and three months post-intervention with German speaking residents, using random digit dialling. There was no change in spontaneous recall of symptoms or signs but when presented with a list (non-spontaneous recall) there was a significant increase in the proportion who, could correctly identify 'paresis or weakness' as a stroke symptom (p < 0.01). There was little change in those who would respond to a stroke as an emergency, albeit with a high baseline (82%). This was the most comprehensive campaign using a greater variety of media than the other studies, but it is not possible to determine which component had the greatest impact. Respondents were asked which information source they remembered but this may not have been the medium that was effective in increasing awareness. A strength of this study was the use of both spontaneous and non-spontaneous recall, as it may be argued that the former may underestimate and the latter may overestimate levels of knowledge; the authors could compare the responses using both methods.

In Ontario, Canada, Hodgson et al. [27] assessed the impact of two television advertising campaigns, of nine and eight months' duration, conducted by the Heart and Stroke Foundation. The campaigns were aimed at people age 45 years and older and illustrated five stroke warning signs (weakness, trouble speaking, vision, headache and dizziness) with an overlaying stamp reading "sudden". A voiceover encouraged viewers to call 911 or their local emergency number if they experienced any of the symptoms. Six telephone surveys were conducted with a sample of Ontarians aged 45 and over using random digit dialling. These were carried out: two months prior to, during and immediately following the first campaign; and during, immediately following and six months after the second campaign. The team also recorded emergency department visits from three months pre- to six months post-intervention (31 months). There was an increase in those who could name two or more symptoms (p < 0.001) and in the mean number of symptoms named (p < 0.001). However, six months after the end of the second campaign, there was a decrease in those who could name two or more symptoms and in the mean number of symptoms named (p < 0.001). There was also a significant increase in the mean number of emergency department visits for stroke and TIA in campaign months compared to non-campaign months (p < 0.01), but the individual contribution of the campaign to this in a regression model for stroke was small, explaining 9% of the variance for total visits, 15% for visits within five hours, 5% for visits within 2.5 hours, although 30% for TIAs. Due to the decrease in awareness of symptoms in the five month period following the end of the intervention the authors conclude that continuity of exposure to the campaign is important in maintaining and increasing stroke awareness.

Fogel et al. [29] conducted a study in Missoula County, USA of television, radio and newsprint advertisements targeting residents aged 45 and over. There were four different television advertisements: warning signs and the need for an emergency response; a three step test if a stroke is suspected; stroke risk factors; and what happens to the brain when treatment is delayed. Radio and newsprint advertisements contained messages on stroke signs, the stroke test and the need for an emergency response. The campaign ran for two 10 week periods, three months apart. Telephone surveys were conducted before and after the campaign (times not specified) using random digit dialling with residents aged 45 and over. There was a significant increase in the mean number of correctly identified symptoms (p < 0.05), and the ability to name two or more in both women and men, and in those aged 45 and over (total 84% after compared to 67% before). There was a significant increase (p < 0.05) in those reporting that they would call emergency services if they experienced sudden speech problems (p < 0.05), numbness or loss of sensation (p < 0.05) or paralysis that did not go away (p < 0.05) but not if they witnessed a stroke.

### Interventions targeted at both the public and health professionals

Four papers evaluated studies where the public and health care professionals were both targeted [32-35]. Two studies were conducted as part of recruitment strategies in clinical trials of thrombolysis [32,33]. Only one study used a controlled before and after design [34],

#### Controlled before and after studies

In five East Texas counties, Morgenstern et al. [34,36] implemented two interventions of 15 months' duration to increase the proportion of patients treated with thrombolysis following licensing of rtPA in the USA. The community intervention consisted of billboard advertising, radio and television public service announcements, news stories, brochures and posters. Also, volunteers were trained in stroke recognition and appropriate action; these volunteers then trained others. The intervention did not appear to target any specific group. A comparison control community was selected with hospitals matched with the intervention community and with similar demographic characteristics but in a different media area. The health professional intervention consisted of news stories and newsletters, highlighting successes and accomplishments in stroke treatment. In addition hospital teams developed emergency department protocols, scheduled continuing medical education and mock 'stroke codes' for staff. Outcome measures were rtPA treatment rates and time to presentation, and data were collected at baseline and during the interventions.

The use of rtPA increased in all patients in the intervention community (p = 0.01) with no change in the control (p = 1.00). There was no change in time to hospital presentation that could be ascribed to the intervention. Time to presentation to hospital decreased in both communities, yet treatment rates increased only in the community receiving the intervention. This would suggest that the main impact may have been on professionals rather than on the public. However, it may be that the community mass media campaign, which was inextricably linked to the professional campaign, also contributed to the professional behaviour change, though this is not possible to disentangle. A later study showed no significant increase in presentation within two hours of onset, but did show increases in rtPA treatment rates that were sustained beyond the intervention, suggesting that the impact was on professional awareness and service organisation rather than public or patient awareness, despite a well developed public campaign[36]

#### Uncontrolled before and after studies

In Durham, North Carolina, USA Alberts et al [32] evaluated the impact of interventions conducted to improve recruitment to a trial of t-PA (tissue-type plasminogen activator). To target the community, features were broadcast on television and radio where the use of t-PA was discussed, the need for early treatment was emphasised and stroke symptoms were described. Newspaper articles were published covering the same points. For health professionals, the intervention consisted of presentations about the t-PA study by specialists in local and regional hospitals and at medical group meetings, and letters (with the study protocol) sent to local and regional physicians. The interventions were of three months' duration. The outcomes of interest were time of presentation to hospital and data were collected from 14 months pre- to 12 months post-intervention. There was an increase in the number of patients with cerebral infarction who presented to hospital within 24 hours of symptom onset in the 12 months after the campaign (p < 0.00001). This study was conducted over 10 years ago before the widespread availability of t-PA and they do not report on presentation within the early hours following stroke.

Barsan et al's [33] study, linked to the NIH Tissue Plasminogen Activator Pilot Study, evaluated a public campaign and professional educational programme in three US states. The public campaign focused on stroke symptoms and the need for an emergency response, and entailed public service announcements and interviews broadcast on television and radio and in newsprint. In relation to health professionals, each participating centre developed an educational programme. These included informational mailings, training programmes and educational lectures. The duration of the interventions were not specified. Outcome data on time to presentation and number of stroke related emergency calls were collected for 30 months from the time the intervention commenced. They found a significant decline in the mean time of presentation to hospital during the intervention (p < 0.05), but no change in time to first medical contact. The authors argue that use of emergency telephone call was the most likely explanation for this; although this varied across sites. Those travelling by ambulance arrived earlier than those using other transport. A number of hospitals joined the project during the measurement period complicating analysis and interpretation. Again this study was conducted over 10 years ago before the widespread availability of rt-PA but they are able to report on time to presentation from under one hour to 24 hours.

In Houston, Texas, Wojner-Alexandrov et al. [35] assessed the impact of a 12 month community and health professional education intervention. In the community intervention television, radio and newsprint were used to convey information on the identification of stroke warning signs and the designation of stroke centres. In addition community stroke screening events were held. No target group within the community was specified. For professionals, monthly education sessions were held for paramedic and hospital staff; also the Los Angeles Pre-Hospital Stroke Scale was implemented with paramedics for use in diagnosing stroke. For six months prior to and for the duration of the intervention, paramedic diagnostic accuracy, time of presentation to hospital and thrombolysis rates were measured. There was an increase from 74 patients admitted per month to 89 per month during the intervention phase (p < 0.001). Paramedic diagnostic accuracy also increased (statistics were not reported). Transport times significantly increased (from 42.2 to 45.8 minutes, p < 0.01), as a result of increased time spent on scene and time from scene to hospital, but overall the median increase was only 3.6 minutes. Impact on treatment rates was inconsistent across hospitals. Prior to the intervention, two of the six study hospitals did not treat any strokes with rtPA; afterwards all hospitals did so and treatment rates ranged from 6.8% to 17.2%. During the intervention four hospitals increased the number of rtPA treatments - in only one was this statistically significant (p = 0.047) - and in two there was a reduction.

### Review of intervention development using the MRC Framework

None of the studies reported that the intervention had an identified theoretical base. For the majority there was no description of intervention development and no mention of any modelling and exploratory or pilot work to test the processes [26-30,32,33,35]. A number selected the design as they believed it had been effective in earlier campaigns of stroke education [28,30] or followed on from previous work [27,31]. Only one study had conducted any developmental work prior to the launch of the campaign [34]; in Phase 1 of the study, focus groups with stroke patients and carers and a telephone survey of the general population were conducted to identify factors that might delay hospital presentation; these study authors also created a community advisory board to support intervention development.

## Discussion

This review has focused on evaluations of mass media interventions designed to increase public recognition of stroke symptoms, the emergency response to stroke and early intervention. This is particularly pertinent when national mass media campaigns, such as the stroke awareness campaign (FAST) in England [20], are being funded and widely implemented.

### Public only interventions

Included studies evaluating public only interventions used a range of methods within their mass media campaigns. Out of the six studies only two had a control group [30,31]; therefore it is not possible to determine if any changes were due to the campaign in the uncontrolled studies. Only two employed a representative population sampling strategy to evaluate impact [27,30]. Effectiveness was evaluated by telephone surveys, thereby excluding members of the community without access to, or unable to use, a telephone, possibly older people and those with communication difficulties. Only one of the studies collected data on stroke admissions and time to presentation [27]; the rest were not able to demonstrate whether increased knowledge translated into the appropriate action on witnessing or experiencing a stroke. All used free recall of stroke symptoms which may underestimate the level of awareness; however one study also used assisted recall and found increased knowledge of only one specific symptom post-intervention [28]. Some campaigns may have been contaminated by other low level stroke education, such as public service announcements, making it difficult to attribute any changes to the intervention [26,28-30].

As stated earlier, only two studies used a more robust design and included a control arm. Unfortunately, one was underpowered and did not target or measure awareness of the need for an emergency response [30]. The other controlled study was a repeat of an earlier campaign [29] in a different county but with the addition of distributed written educational materials and a control county [31]. The second controlled campaign revealed a lower percentage point increase in awareness of stroke symptoms and intention to call emergency services if they experienced or witnessed specific stroke symptoms than in the first uncontrolled study (Additional File 3). As there were no significant changes in the control community it can be concluded that any increases demonstrated, albeit modest, were more likely to be as a result of the intervention.

Data on the longer term retention of stroke knowledge of symptoms and signs was poor in most of the studies. Only one study collected outcomes six months after the end of the campaign to assess longer term impact and found that knowledge declined post- intervention [27]. It is impossible to assess the longer term impact, or to know whether or when an intervention should be repeated, unless evaluations measure impact at a range of time points following intervention.

Overall, the impact of these interventions was inconsistent. Nonetheless, all showed some significant increase in awareness of symptoms but little impact on the awareness of the need for, or intention to call, an emergency response, although baseline levels were high. In view of the fact that these campaigns have failed to increase awareness of the need for an emergency response to stroke [26,28,29,31] or prompt those experiencing a stroke to present at hospital immediately [27] further research is warranted to explore people's values and beliefs in relation to stroke.

### Public and professional interventions

The public and professional campaigns largely focused on outcomes of time to hospital and thrombolysis rates [32-35]. Measures of time to arrival varied across studies. Two of the studies were over 10 years old and were part of a drive to increase recruitment into trials of r-PA and most of the professional intervention appeared to be aimed at raising awareness of the trial.

Given the complexity of interventions and the dual nature (public and professional) of these four studies it is very hard to disentangle any active components that might explain any reported impact. This is a feature noted in the Cochrane systematic review evaluating mass media campaigns designed to increase health service utilisation, incorporating 15 interrupted time series studies [17]; nonetheless this concluded that "those engaged in promoting better uptake of research information in clinical practice should consider mass media as one of the tools that may encourage the use of effective services and discourage those of unproven effectiveness". As with our conclusions specific to stroke, the authors stated that it was "difficult to determine extent [of change] attributable to changes in health care providers or consumers" given that many targeted both public and professionals.

### Intervention design

Despite the importance of a theoretical base and early stage research in the design of the intervention [18] only one study described conducting exploratory work to identify factors that may have influenced people from seeking emergency medical care at the onset of stroke symptoms [34]. Nevertheless the findings from that study indicate that any increase in thrombolysis rates were most probably due to the component of the intervention targeting health professionals rather than those in the community. Had the team carried out an exploratory trial to test the intervention in the community they may have been able to refine it accordingly and influence time to presentation at hospital.

A few conducted their community campaigns based on the results of earlier ones [28,30] even when the impact on the community was not determined [34] and any improvement in awareness of stroke symptoms was not measured beyond one month post-intervention [26].

## Limitations

There are limitations to this review as a result of the range of methods and study designs employed in the included studies. Only three studies used a controlled before and after design [30,31,34], limiting the conclusions that can be drawn. Of the ten studies only the latter three would be accepted as valid for inclusion in a systematic review by the Cochrane Effective Practice and Organisation of Care Group [37]. Therefore it is not possible to state with any degree of confidence whether any changes identified were attributable to the intervention or to other factors. Few studies had pre-specified primary outcome measures and several used multiple analyses. None of the studies used qualitative methods (such as interviews with participants) to help understand their findings [38]. There was very little reporting of the theoretical or empirical basis of interventions, or of their development methods, as recommended by the MRC Framework for development and evaluation of complex interventions; it appears that few studies have built upon earlier, potentially promising, interventions.

One published study not included in this review reported the development and piloting of an educational kit (Stroke Heroes Act FAST animation, brochure and poster) designed to improve knowledge of the symptoms of stroke and the need for emergency action using the FAST acronym [39]. The materials from this educational kit have been used in 28 countries. In Massachusetts they have been used in mass media campaigns and evaluated through telephone surveys [40] and claim to have significantly improved knowledge of symptoms and the need for emergency response, but these results do not appear to have been published in peer reviewed journals.

## Conclusions

In conclusion, although some studies showed increases in symptom awareness and awareness of need for emergency response, and increased use of emergency transport, none show a full picture of increased awareness, increased use of emergency response, shorter time to arrival and increased use of thrombolysis following a mass media campaign. There is clearly a need for more robust evaluation of such campaigns using studies with at least a controlled before and after design, and including qualitative methods to support understanding of any demonstrated impact and to help unpick the elements of any campaign that might be important. The nationwide campaign in England implemented in February 2009 offered an opportunity to do this, but unfortunately there is no such robust evaluation in progress, despite considerable investment of public resources [20]. The Department of Health website reports a 55% increase in emergency calls for stroke following campaign implementation but no further details are provided [41]. When campaigns are evaluated, such as those using the Stroke Heroes Act FAST materials [40], there is a need for the results to be published in peer reviewed journals. This would inform others of the efficacy of the intervention and robustness of the evaluation, and add to the existing body of knowledge about improving response to stroke.

Finally, the reported studies not only have limited methodological evaluation, but also little evidence of theoretically grounded development and piloting of the interventions. With the exception of the Morgenstern et al. study [34], there is no evidence of such structured development. Mass media campaigns clearly can be successful in improving knowledge and changing behaviours in other fields of health and safety promotion. Unlike other interventions, such as stroke patient education and community stroke screening programmes, mass media campaigns have the potential to improve knowledge and awareness and change the behaviours of a large number of people. We would urge future developers of mass media campaigns to consider a more structured approach, such as that recommended in the MRC Framework for the Development and Evaluation of Complex Interventions [23].

## Competing interests

Authors JL, MM, HR, MW and RT have no competing interests. GF has consultancy relationships with Boehringer Ingelheim and Lundbeck, grants/grants pending from Servier Pharmaceuticals, Lundbeck and Mitsubishi, payment for development of educational presentations including service on speakers' bureaus from Boehringer Ingelheim and travel/accommodations expenses covered or reimbursed by Boehringer Ingelheim that might have an interest in the submitted work in the previous 3 years.

## Authors' contributions

JL conducted the searches and electronic sifting of titles and abstracts. JL and HR independently reviewed the retained papers and extracted data. JL and RT wrote the first draft of the manuscript. All authors commented on the first draft and all revisions.

## Pre-publication history

The pre-publication history for this paper can be accessed here:

http://www.biomedcentral.com/1471-2458/10/784/prepub

## Supplementary Material

Additional File 1 **Search Terms for Medline**. Click here for file

Additional File 2 **Characteristics of Included Studies**. Click here for file

Additional File 3 **Results of Evaluations**. Click here for file
