# Supplementary material for: Systematic review of mass media interventions designed to improve public recognition of stroke symptoms, emergency response and early treatment
Source: BMC Public Health. 2010 Dec 23;10:784. doi: 10.1186/1471-2458-10-784 (PMC3022856; doi:10.1186/1471-2458-10-784)
Supplement: Additional File 1 — Search Terms for Medline. [file 1471-2458-10-784-S1.DOCX]

**Table 1 Search terms for Medline (Cochrane search terms for stroke 1-90)**

1. cerebrovascular disorders/
2. exp basal ganglia cerebrovascular disease/
3. exp brain ischemia/
4. exp carotid artery diseases/
5. cerebrovascular accident/
6. exp brain infarction/
7. exp cerebrovascular trauma/
8. exp hypoxia-ischemia, brain/
9. exp intracranial arterial diseases/
10. intracranial arteriovenous malformations/
11. exp "Intracranial Embolism and Thrombosis"/
12. exp intracranial hemorrhages/
13. vasospasm, intracranial/
14. vertebral artery dissection/
15. aneurysm, ruptured/
16. brain injuries/
17. brain injury, chronic/
18. exp carotid arteries/
19. endarterectomy, carotid/ or endarterectomy/
20. *heart septal defects, atrial/
21. *atrial fibrillation/
22. (stroke or poststroke or post-stroke or cerebrovasc$ or brain vasc$ or cerebral vasc$ or cva$ or apoplex$ or isch?emi$ attack$ or tia$1 or neurologic$ deficit$ or SAH or AVM).tw.
23. ((brain$ or cerebr$ or cerebell$ or cortical or vertebrobasilar or hemispher$ or intracran$ or intracerebral or infratentorial or supratentorial or MCA or anterior circulation or posterior circulation or basal ganglia) adj10 (isch?emi$ or infarct$ or thrombo$ or emboli$ or occlus$ or hypox$ or vasospasm or obstruction or vasculopathy)).tw.
24. ((lacunar or cortical) adj5 infarct$).tw.
25. ((brain$ or cerebr$ or cerebell$ or intracerebral or intracran$ or parenchymal or intraventricular or infratentorial or supratentorial or basal gangli$ or subarachnoid or putaminal or putamen or posterior fossa) adj10 (haemorrhage$ or hemorrhage$ or haematoma$ or hematoma$ or bleed$)).tw.
26. ((brain or cerebral or intracranial or communicating or giant or basilar or vertebral artery or berry or saccular or ruptured) adj10 aneurysm$).tw.
27. (vertebral artery dissection or cerebral art$ disease$).tw.
28. ((brain or intracranial or basal ganglia or lenticulostriate) adj10 (vascular adj5 (disease$ or disorder or accident or injur$ or trauma$ or insult or event))).tw.
29. ((isch?emic or apoplectic) adj5 (event or events or insult or attack$)).tw.
30. ((cerebral vein or cerebral venous or sinus or sagittal) adj5 thrombo$).tw.
31. (CVDST or CVT).tw.
32. ((intracranial or cerebral art$ or basilar art$ or vertebral art$ or vertebrobasilar or vertebral basilar) adj5 (stenosis or isch?emia or insufficiency or arteriosclero$ or atherosclero$ or occlus$)).tw.
33. ((venous or arteriovenous or brain vasc$) adj5 malformation$).tw.
34. ((brain or cerebral) adj5 (angioma$ or hemangioma$ or haemangioma$)).tw.
35. carotid$.tw.
36. (patent foramen ovale or PFO).tw.
37. ((atrial or atrium or auricular) adj fibrillation).tw.
38. asymptomatic cervical bruit.tw.
39. exp aphasia/ or anomia/ or hemiplegia/ or hemianopsia/ or exp paresis/ or deglutition disorders/ or dysarthria/ or pseudobulbar palsy/ or muscle spasticity/
40. (aphasi$ or apraxi$ or dysphasi$ or dysphagi$ or deglutition disorder$ or swallow$ disorder$ or dysarthri$ or hemipleg$ or hemipar$ or paresis or paretic or hemianop$ or hemineglect or spasticity or anomi$ or dysnomi$ or acquired brain injur$ or hemiball$).tw.
41. ((unilateral or visual or hemispatial or attentional or spatial) adj10 neglect).tw.
42. or/1-41
91. intervention studies/

92. public health campaign.mp. or campaign.mp.

93. education$ program$.mp.

94. 91 or 92 or 93

95. 42 and 94

96. Limit 95 to (english language and humans and yr="1980-2010")
