# Supplementary material for: Systematic review of mass media interventions designed to improve public recognition of stroke symptoms, emergency response and early treatment
Source: BMC Public Health. 2010 Dec 23;10:784. doi: 10.1186/1471-2458-10-784 (PMC3022856; doi:10.1186/1471-2458-10-784)
Supplement: Additional File 2 — Characteristics of Included Studies. [file 1471-2458-10-784-S2.DOCX]

**Table 2 – Characteristics of included studies**

| **Multi-media campaigns**  ***Public only*** | | **Duration of intervention** | **Design, Outcomes, Outcome measure** | **Measurement timepoints** | |
| --- | --- | --- | --- | --- | --- |
| **Author** | **Intervention** |  |  | **Pre-** | **Post** |
| Becker 2001  USA | The educational intervention consisted of public service announcements (PSAs) utilizing several different types of media dominated by TV and press and with particular emphasis on calling 911. 1. Television - three PSAs depicting different signs and symptoms of stroke aired during May and August 1998 on ABC. Stroke PSAs produced by other organisations and several public interest stories about stroke were also aired during May 1998. UWTV produced an hour long segment on stroke which was aired over 10 times during the summer of 1998. 2. Newspaper - Six one half page advertisements were purchased in city newspapers in May and August 1998 (Wed and Sun editions). Ads focused on risk factors for and the signs and symptoms of stroke and the need to call 911. Stroke interest stories (translated into 5 other languages) also appeared in community newspapers distributed to the city's low income population and to the elderly population. 3. Miscellaneous - 17 public stroke screenings focused on ethnic minorities were conducted throughout the region during the course of the campaign. 'Brain attack' fliers which highlighted the risk factors for stroke were distributed.  **Message**: Warning signs. Call 911 | 5 mths | Uncontrolled before & after  Stroke knowledge inc:  Action on witnessing a stroke  Risk factors  Signs & symptoms  Telephone survey at two time points only | 1 mth | 1 mth |
| Silver 2003  Canada | Mass media campaigns using three different media strategies in three separate areas: a) Continuous high level TV advertising, b) intermittent TV advertising and c) newspaper ads. 30 second black and white telephone advertisement giving the warning signs of stroke and a print advertisement based on the television ad. Also a control group (had only Heart and Stroke Foundation Public Service Announcements available to all media across the province)  .**Message:** Warning signs | 18 mths | Controlled before & after  Ability to name two or more symptoms  Telephone survey | Immediately | 3 mths |
| Hodgson 2007  Canada | Two television advertising campaigns conducted by the Heart & Stroke Foundation of Ontario. The 2003/2004 campaign was of 9 months duration and the 2005 campaign for 8 months. The advertisement illustrated the key words of the five stroke warning signs (weakness, trouble speaking, vision, headache and dizziness) with an overlaying stamp reading "sudden". It concluded with a call to action in the form of a voiceover encouraging viewers to call 911 or their local emergency number if they experienced any of these symptoms.  **Message**: Warning signs. Call 911 | 9 mths  8 mths | Uncontrolled before & after  No of warning signs  Ability to name ≥2 symptoms  ED presentations for stroke  Telephone survey & hospital records (stroke register over 11 stroke centres) | 2 mths | During & at end of both campaigns. 6 mths later |
|  |  |  |  | ED visits recorded for 31 mths (4 mths pre to 6 mths post) | |
| Marx 2008  Germany | The intervention comprised of public announcements in form of poster advertisements, flyers, mail circular, slogans, stroke interest stories etc in local newspapers, on television and radio and public events.  **Message**: Warning signs. Call emergency | 3 mths | Uncontrolled before & after  Stroke knowledge: General knowledge on stroke eg occurs in brain, age of occurence  Risk factors  Signs & symptoms  Action on witnessing a stroke  Telephone survey | 3 mths | 2 mths |
| Fogle 2008  USA | The campaign included four television and three radio advertisements that addressed the warning signs for stroke and the need to call 911 and act quickly. One television advertisement addressed stroke warning signs and depicted former stroke patients who survived because their spouses knew the stroke signs and called 911. A second television advertisement described a simple three step test that consumers could take if they or someone they witnessed may be having a stroke. The third television advertisement depicted a female physician describing stroke risk factors, and the fourth advertisement focused on brain cell death associated with delays in treatment. The radio and newsprint advertisements contained similar messages on stroke signs, the stroke test, and the need to call 911 immediately. Newsprint advertisements were placed in the community newspaper each Sunday during the 20 week period.  **Message**: Warning signs. Call 911 | 2 x 10 week periods | Uncontrolled before & after  Awareness of stroke warning signs & the need to call 911  Telephone survey at two time points only | Before first period† | After second period† |
| Fogle 2010  USA | The campaign materials appear to be a replication of those used in the earlier campaign (Fogle et al 2009). Educational materials were posted to community doctors and pharmacies, churches and care homes for older people; a stroke information brochure and magnet were posted to households with residents in the target age group.  **Message**: Warning signs. Call 911 | 2 x 10 week periods | Controlled before & after.  Awareness of stroke warning signs & the need to call 911  Telephone survey at two time points only | Before first period† | After second period† |

| ***Public & professionals*** | **Public** | **Professional** | **Duration of intervention** | **Design, Outcomes, Outcome measure** | **Pre Post** | |
| --- | --- | --- | --- | --- | --- | --- |
| Alberts 1992  USA | Local and regional television and radio stations presented features dealing with the use of t-PA for acute stroke and the need for early treatment. These stories featured interviews with patients and physicians and highlighted the symptoms of stroke and TIA. Regional radio stations conducted interviews and call-in talk shows that emphasised the need for rapid recognition and evaluation of stroke patients. Articles appeared in local newspapers describing stroke symptoms, the t-PA study and the time limitations for treatment. **Message**: rTPA for stroke & the need for early administration. Also symptoms of stroke/TIA | Education of physicians and other health providers was primarily about the existence of the rtPA study and consisted of lectures and mass mailings to local doctors as well as changes in service delivery, including more-on-call neurology staff and use of a patient transfer helicopter. Team of neurologists with special training and interest in CVD spoke at the study hospital, local and regional hospitals, ERs and other medical group meetings. | 3 mths | Uncontrolled before & after  Time of presentation to hospital  Hospital documentation | 14 mth period | 12 mth period |
| Barsan 1994  USA | Multi-media with PSA and interviews on radio, television and newspaper concentrating on the signs and symptoms of stroke and the need to call 911.  **Message**: Signs & symptoms. Call 911 | Educational programmes for professionals included: informational mailings, in service training programmes and educational lectures. This was targeted at emergency department medical and nursing staff, wider hospital medical staff, and paramedics and pre-hospital care providers. | Not reported | Four post-intervention quartiles  Time of presentation to hospital  Hospital documentation | N/A | Records screened from start of intervention for 30 mths |
| Morgenstern 2002  USA | Community intervention - Mass media strategies included billboard advertising, radio and television public service announcements and news stories. "Small" media included brochures and posters. Over 600 volunteers were trained in stroke recognition and reaction. Community figures were used as role models to show calling 911 immediately for stroke symptoms and to demonstrate that responding to stroke immediately can result in a better outcome. Community members were also encouraged to be assertive in asking the physician about TPA if taken to the ED for stroke symptoms, thereby providing a cue to act for healthcare providers who were initially reluctant.  **Message**: Stroke recognition, calling 911 can result in better outcome & ask about TPA | The healthcare provider component of the intervention relied on systems change in hospitals, change of perceived norms in the medical community and reinforcement of behaviour change through both mass media (e.g. highlighting successes in news stories) and small media (e.g. providing newsletters to primary care providers, emergency physicians, and neurologists that provided feedback regarding stroke treatment accomplishments). Multidisciplinary teams in hospitals developed emergency department protocols, solved problems related to responsibility for care and scheduled continuing medical education and mock "stroke codes" for both hospital and EMS staff. | 15 mths | Quasi-experimental (control not randomly selected, but matched hospitals and demography)  Thrombolytic rates  Hospital documentation - blinded | Baseline | During  (15 mths) |
| Wojner-Alexandrov 2005  USA | Public announcement of stroke centre designation; multi-media community education regarding identification of stroke warning signs using print, radio and televised media, community stroke screening events.  **Message**: Recognition of stroke warning signs | Monthly education sessions for paramedic & hospital staff with comparative benchmarking of hospital and paramedical performance, and Implementation of the Los Angeles Pre-Hospital Stroke Scale by the Fire Department. | 12 mths | Uncontrolled before & after  Paramedic diagnostic accuracy  Time of presentation to hospital  Thrombolytic rates  Hospital & paramedic records | 6 mth period | During intervention (12 mths) |

† specific times not given
