# Supplementary material for: Systematic review of mass media interventions designed to improve public recognition of stroke symptoms, emergency response and early treatment
Source: BMC Public Health. 2010 Dec 23;10:784. doi: 10.1186/1471-2458-10-784 (PMC3022856; doi:10.1186/1471-2458-10-784)
Supplement: Additional File 3 — Results of Evaluations. [file 1471-2458-10-784-S3.DOCX]

**Table 3 – Results of evaluations**

| **Multi-media campaigns**  ***Public*** | | |  |  |  |  |  |
| --- | --- | --- | --- | --- | --- | --- | --- |
| **Study** | **Inclusion/exclusion criteria** | **Sample** | **Participants/ cases n=** | **Basic demographic** | **Outcome** | **Results** | |
| Becker 2001 | English speaking residents | Independent at each time-point | 547 pre-test | 55% <45; 56% white; 47% female, 22% high school or less | Aware of ≥1 stroke symptom  Awareness of emergency response (What would you do first?) | 7% point increase (p=0.032). 38.6% to 45.6%  4% point decrease (ns) 68.2% to 64.0% | |
|  |  |  | 511 post-test | 53% <45; 54% white; 43% female, 24% high school or less |  |  |  |
| Silver 2003 | Not specified | Independent at each time-point | I=1109 pre-  I=1209 post-  C=405 pre-  C=410 post | Not reported | Name ≥2 warning signs  Mean number of warning signs | high intensity TV 14% point increase (p<0.001) 40.4% to 54.1%  low intensity TV 11% point increase (p=0.002) 38.8% to 49.6%  print 41.7% to 40.8% (p=0.8)  control 43.7% to 35.9% (p=0.022)  high intensity TV 1.32 to 1.66 (p<0.001)  low intensity TV 1.27 to 1.47 (p=0.021)  print 1.25 to 1.17 (p = 0.280)  control 1.38 to 1.10 (p = 0.001) | |
| Hodgson 2007 | Not specified | Independent at each time point | 1000+ at each of the 6 surveys | All > 45 yrs of age | Name ≥2 warning signs  Mean number of warning signs  Time to presentation | 20% point increase (p<0.001) 52.1% to 72.3%. 6 mths post campaign dropped to 63.6%  1.69 to 2.31(p<0.001). 6 mths post campaign dropped to 1.99.  Whilst there was a significant increase in mean number of ED visits for stroke the proportion within 5 and 2.5 hrs was not apparently affected during campaign periods | |

| **Multi-media campaigns**  ***Public*** | | |  |  |  |  |  |
| --- | --- | --- | --- | --- | --- | --- | --- |
| **Study** | **Inclusion/exclusion criteria** | **Sample** | **Participants/ cases n=** | **Basic demographic** | **Outcome** | **Results** | |
| Marx 2008 | German speaking residents | Independent at each time point | 507 pre-test | Mean age 53.1 yrs; 57% female, 40% college or higher | Identified and prompted ‘paresis /weakness’  Awareness of emergency response | This was the only significant finding of a number of symptoms tested: 7% point increase (p<0.01) 88%-95%  Also those correctly identifying that stroke can occur “at any age” rose from 45% to 62% (p<0.005)  1% point increase from 81% to 82% (ns) | |
|  |  |  | 501 post-test | Mean age 51.6 yrs; 56% female, 40% college or higher |  |  |  |
| Fogle 2008 | Adults aged 45 yrs & older resident in study area were included | Independent at each time-point | 400 pre-test  400 post-test | 35% ≥ 65 yrs; 63% female; 93% education ≥ 12 yrs  38% ≥ 65 yrs; 59% female, 95% education ≥ 12 yrs | Name ≥2 warning signs  Mean number of warning signs  Awareness of emergency response  **Would call 911 if experienced:**  Paralysis  Numbness  Speech problems | 17% point increase (p<0.05) 67 to 84%  1.82 to 2.25 p<0.05  2% point increase (ns) - 74% to 76%  16% point increase (p≤0.05) 42% to 58%  10% point increase (p≤0.05) 41% to 51%  7% point increase (p≤0.05) 51%to 58% | |
| Fogle 2010 | Adults aged 45 yrs & older resident in study area were included | Independent at each time-point | I= 400 pre-  I= 400 post- | 39% ≥ 65 yrs; 65% female; history of: AF 15%; high BP 43%; heart disease 12% | Name ≥2 warning signs    Awareness of emergency response  **Would call 911 if experienced:**  Numbness  Any of 3 symptoms (speech problems, numbness  or paralysis) | 9% point increase (p≥0.05) 73 to 82%  3% point increase (ns) - 81% to 84%  6% point increase (p≤0.05) 50% to 56%  9% point increase (p≤0.05) 39%to 48%  No significant change in control community | |
|  |  |  | C= 400 pre-  C= 400 post- | 32% ≥ 65 yrs; 57% female; history of: AF 9%; high BP 31%; heart disease 7% |  |  |  |

| ***Public & professionals*** | | |  |  | |  |  |  |
| --- | --- | --- | --- | --- | --- | --- | --- | --- |
| **Study** | | **Inclusion/exclusion criteria** | **Sample** | **Participants/ cases n=** | | **Basic demographic** | **Outcome** | **Results** |
|  |  | |  |  | |  |  |  |
| Alberts 1992 | Ptts with cerebral infarction included. Those with in-hospital strokes or TIA were excluded from analysis | | Independent at each time-point | 290 pre-intervention  189 post intervention | | Not reported | Admitted within 24 hours of symptom onset | 49% increase (p=0.0001), 37% to 86% for those with cerebral infarction |
| Barsan 1994 | Data were gathered only on those presenting within 24 hrs of stroke onset | | N/A | 1948 (time data available for 1116) | | 17% <55 yrs; 76% white; 59% female | Mean time from symptom onset to hospital arrival  Called emergency | 1.7 hr mean decrease from 1^st^ to 4^th^ quartile, 3.2 to 1.5hrs (p<0.32)  21% point increase from 1^st^ to 4^th^ quartile, 39% to 60% |
| Morgenstern 2002 | All cerebrovascular patients admitted to control or intervention community hospitals | | N/A | baseline | I=218 | Mean 73 yrs; 57% female, 77% white | Treatment rates (rTPA)  Arriving within 2 hours of symptom onset | % point increases in  All cerebrovascular patients 3.95%, 1.38% to 5.75% (p=0.01)  Ischaemic stroke only 6.44%, 2.21% to 8.65% (p=0.02) rising further to 11.2% 6 months later (Morgenstern 2003)  Eligible patients 38%, 14% to 52% (p=0.003), rising further to 69% 6 months later  (Control community had no significant change)  ns |
|  |  |  |  |  | C=206 | Mean 71 yrs; 62% female, 66% white |  |  |
|  |  |  |  | during | I=400 | Mean 71 yrs; 57% female, 79% white |  |  |
|  |  |  |  |  | C=365 | Mean 73 yrs; 58% female, 72% white |  |  |
| Wojner-Alexandrov 2005 | Cases of suspected acute stroke admitted to study hospitals in Houston. | | N/A | 446 cases pre-  1072 cases during | | Mean 69 yrs; 56% female; 44% white (overall figures given) | Correct paramedic diagnosis  Arriving within 2 hrs of symptom onset  Thrombolysis rates | 18% point increase in positive predictive value from 61% to 79%  4% point increase (p=0.002), 58% to 62%  In 4 centres % point increases of 3.9, 6.8, 8.1 & 12.5. In 2 centres decreases of 3.6% & 6% |
